# Assessing the cadmium phytoremediation potential of different *Solanum lycopersicum* L. cultivars during their vegetative growth phase

**DOI:** 10.3389/fpls.2025.1587253

**Published:** 2025-05-21

**Authors:** Jibao Jia, Huiping Dai, Jie Zhan, Shuhe Wei, Lidia Skuza, Junjun Chang

**Affiliations:** ^1^ Yunnan Key Laboratory for Plateau Mountain Ecology and Restoration of Degraded Environments, School of Ecology and Environmental Science, Yunnan University, Kunming, China; ^2^ College of Biological Science & Engineering, Qinling-Bashan Mountains Bioresources Comprehensive Development C.I.C, State Key Laboratory of Biological Resources and Ecological Environment Jointly Built By Qinba Province and Ministry, Shaanxi Province Key Laboratory of Bio-resources, Shaanxi University of Technology, Hanzhong, China; ^3^ Department of Health Management, Liaoning Vocational College Of Medicine, Shenyang, China; ^4^ Key Laboratory of Pollution Ecology and Environment Engineering, Institute of Applied Ecology, Chinese Academy of Sciences, Shenyang, China; ^5^ Institute of Biology, Centre for Molecular Biology and Biotechnology, University of Szczecin, Szczecin, Poland

**Keywords:** *Solanum lycopersicum* L., *Solanum nigrum* L., phytoremediation, cadmium, cultivar

## Abstract

**Introduction:**

*Solanum nigrum L.* has been a widely studied Cd hyperaccumulator. *Solanum lycopersicum L.* also belongs to the same family (Solanaceae) with *S. nigrum.* Compared to *S. nigrum*, the Cd accumulation characteristics and remediation potentials of seven S. lycopersicum cultivars at their maximum vegetative growth period stages (MVGPS) were determined. The advantage of the remediating method at MVGPS is to avoid Cd entering food chain via fruits at mature period.

**Methods:**

Soil pot experiments were conducted and used to determine Cd concentration in all test plants. Biomasses and some physiology index were measured either.

**Results:**

The results showed that the enrichment factors (EFs, the Cd concentration ratio of shoots to soils) of the tested *S. nigrum* and all cultivars were greater than 1. However, only the translocation factors (TFs, the Cd concentration ratio of shoots to roots) of *S. nigrum* and Baishite (*S. lycopersicum* cultivar) were higher than 1, indicating that only Baishite exhibited the main properties of a Cd hyperaccumulator. In particular, the Cd concentration (20.27 mg kg-1) in shoot of Baishite was same as that of *S. nigrum*, and they were the highest. Cd shoot accumulation capacity (96.1 µg plant-1) of Baishite was 17.6 % higher than *S. nigrum* due to its higher shoot biomass (an increase of 10.4 %). The shoot of biomass (g plant-1) in Baishite was higher than in *S. nigrum*. Furthermore, some index like chlorophyll content and SOD activity of Baishite were higher either, which might be part reasons for Baishite highly accumulated Cd, but other mechanisms could play more important roles.

**Discussion:**

These findings suggested that some *S. lycopersicum* cultivars could have a higher Cd remediation potential, and further screening for more ideal remediation plants from crop was possible.

## Introduction

With the continuous human demand for resources and the increasing production intensity, wastes containing heavy metals are constantly found in the environment, and the rate of pollution to the environment is still increasing (Ogugua et al., 2022). Among them, Cd pollution in the soil environment is becoming more and more serious, and it is harmful to people and animals (Janifer et al., 2021; Dai et al., 2022). Under this background, phytoremediation of soil heavy metal pollution has gradually become a hot research topic at home and abroad (Liu et al., 2023). Phytoremediation technology is considered to be a green, cheap, and non-interference *in situ* remediation technology. In order to promote phytoremediation efficiency, enhanced hyperaccumulator biomass and shortened remediation cycle are needed (Dai et al., 2022; Dou et al., 2023). *Solanum nigrum* L. is a widely studied Cd hyperaccumulator. Wu et al. (2020) summarized its remediation potential, higher heavy metal accumulation capacities, and some strengthened measurements like suitable microbes and integrated agricultural practices. *Solanum lycopersicum* L. also belongs to Solanaceae just as *S. nigrum.* Some cultivars of *S. lycopersicum* were with higher biomasses. Thus, their remediation potentials may be higher if they owned Cd hyperaccumulator characteristics like *S. nigrum.*

In fact, there were too many studies concerning *S. lycopersicum* heavy metal accumulation. In general, the research scope can be roughly divided into the following four categories. The first category concerned the metals’ effects on physiological indices. Akinc et al. explored the effects of Pb on *S. lycopersicum* growth as well as chlorophyll and water content (Akinci et al., 2010). Wang et al. determined the toxic effect of excess Cu and Zn on some *S. lycopersicum* antioxidant enzymes (Wang et al., 2022). Sarwar et al. determined the influence of Cd on the anatomy of internodes and leaves of *S. lycopersicum* (Sarwar et al., 2023). Other studies concerned the effect of Pb and Cr on *S. lycopersicum* (Almaroai and Eissa, 2020), estimation of Cd tolerance (Rodríguez-Rodríguez et al., 2023), mechanisms of Cd stress avoidance by Se (Zhang et al., 2022), and association of phytohormones with growth (Sun et al., 2023). Only two *S. lycopersicum* cultivars were compared physiologically and biochemically (Al-Lahham et al., 2007). The second research category on *S. lycopersicum* concerned mainly the reduction of heavy metal toxicity to this plant. Some arbuscular mycorrhizal fungi could alleviate Cd stress in *S. lycopersicum* (Pan et al., 2023). Graft reduced Cd accumulation of *S. lycopersicum* (El-Sappah et al., 2021). Su et al. (2021) showed that the cherry tomato cultivar Hanluzhe has lower cadmium accumulation contents in the leaves as compared to Lvfeicui, and different expression genes were mainly involved in plant hormone signal transduction (Ayhan, 2010), antioxidant enzymes, cell wall biosynthesis, and metal transportation. The studies of El-Sappah et al. (2021) focused on the SlMTP genes that displayed different responses in either *S. lycopersicum* leaf or root treatments by five divalent heavy metals. The reciprocal interaction between endophytic *Curvularia lunata* CSL1 and tomato was revealed through transcriptomics research to reduce Cd toxicity (Asaf et al., 2023), genotoxicity induced by heavy metal in two cultivars (Su et al., 2021) or trico-synthesized silicon nanoparticles, and *Trichoderma* metabolites induced by heavy metal (Hasan et al., 2022; Raja et al., 2023). The fourth category research on *S. lycopersicum* involved heavy metal accumulation differences and the remediation potential of different *S. lycopersicum* genotypes differing in Cd accumulation (Wei et al., 2018). The role of plant growth-promoting rhizobacteria (PGPR) and its potential mechanisms to promote plant growth and enhance plant repair ability have received attention (Bhanse et al., 2022). The same goes for the suitability assessment of *S. lycopersicum* in remediating Zn-polluted soil (Akanbi-Gada et al., 2019) and enhancement of Cr phytoremediation by arbuscular mycorrhiza fungi and *Aspergillus terreus* (Pan et al., 2023). However, there were rarely studies concerning Cd hyperaccumulation characteristics and the remediation potential of *S. lycopersicum*, especially for several cultivar species compared to *S. nigrum*. In the vegetative growth period, *S. lycopersicum* gradually adapts to Cd in the seedling stage, and all physiological indexes basically reach the most vigorous state. Selecting this period can more appropriately reflect the maximum accumulation of Cd in tomato plants. Especially in the maximum vegetative growth period, tomato leaves are basically of the same size during the maturity period. Cd mainly accumulates in the stems and leaves. If the plants are harvested during this period and the next remediation process is repeated, it is possible to shorten the remediation cycle and improve the phytoremediation efficiency within a year. At the same time, because no fruit is produced and due to be harvested at the maximum vegetative growth period, without any fruits the food chain risk on human health of heavy metals is also avoided. Therefore, this study has a very important practical significance.

## Materials and methods

### Conduct of the experiment

Mature seeds of *S. nigrum* were collected from an agricultural field in Hanzhong City of China. The seven cultivar seeds of *S. lycopersicum* were procured from the seed markets in Hanzhong and Shenyang cities of China, including Meihao, L-404, Baisite, Zeibutou, Ziyu, Liaoyuanduoli, and L-402.

Top (0–20 cm) soil samples (cinnamon soil) also from the field were used in the pot experiment, with pH 6.82, 0.21 mg kg^-1^ Cd, available potassium content 16.9 mg kg^-1^, organic matter 27.2 g kg^-1^, and available phosphorus 16.7 mg kg^-1^. The soil quality is as follows: 19.4% clay, 40.5% silt, and 32.6% sand. According to NSEQS, the collected soil is pure and therefore considered as control soil (CK). According to the NSEQS standard, polluted soil (PS) was prepared by adding 3.12 mg kg^-1^ of CdCl_2_. Soil was mixed with Cd to a total of 3.09 mg kg^-1^, and each pot was filled with 2.5 kg of treated soil. (NH_4_)_2_SO_4_ was used as fertilizer for each pot (3 g). All test pots were kept for 2 months. After treatment with 0.1% sodium hypochlorite, all plant seeds were put into CK and PS soils. There were three replicates.

This experimental material was cultivated in a greenhouse of the State Key Laboratory of Biological Resources and Ecological Environment of Qinba. The culture parameters include radiation intensity of 1,001 μm m^-2^ s^-1^, temperature of 21°C–28°C, day/night period of 12/12 h, and relative air humidity of 66%. When the seedlings were growing to about 7 cm in height, seven seedlings were preserved. Tap water was supplemented daily. After 70 days at their maximum vegetative growth stages (without flowers), in order to avoid Cd from entering the food chain through fruits during the maturity period, all plants were collected.

Another reason for selecting the maximum vegetative growth stages as the time of harvest is that the main organs of plants accumulating Cd are the leaves and stems of the hyperaccumulator. At the maximum vegetative growth stages of the plants, the biomass values of leaves and stems are nearly the biggest in its whole growth period. Thus, the remediation potential at the maximum vegetative growth stages of the hyperaccumulator is also near the highest (Wei et al., 2009).

### Determination of physical, chemical, and physiological indices

Rhizospheric soils were collected through gentle and manual shaking. According to this method by Dai et al. (2022), the extractable Cd in rhizosphere soil and the total Cd concentration in all samples were determined. GBW07405 (GSS-5) and GBW10015 (GSB-6) standard reference materials were used as QA/QC.

The biomass of harvested plant will be measured by collecting them according to their roots, stems, leaves, and flowers. After drying at 105°C for 5 min, the samples were dried for about 2 days until counterweight under 70°C and weighed (Dai et al., 2021).

The photosynthetic pigment content in plant leaves was determined by Dai et al. (2022). The chlorophyll content was determined as described by Dai et al. (2022). The measurement of SOD activities and MDA contents was based on the methods by Jia et al. (2023). SOD and MDA were determined because they are closely related to a plant’s resistance to heavy metals (Wang et al., 2022; Sarwar et al., 2023).

### Data analysis

SPSS 12 software was used for data processing and analysis. LSD method was used to analyze at the 0.05 level of significance.

## Results

### Cd concentration differences in different parts of plants

**Table 1** indicates that the Cd levels (mg kg^-1^) in *Solanum* roots in the CK treatment were similar (*p* < 0.05). The Cd concentration differences in the stems, leaves, and shoots were not big.

**Table 1:** Cd levels in *Solanum nigrum* and *Solanum lycopersicum* cultivars in control (CK) and polluted soil (PS) treatments.

*Solanum* species and variety	Control (mg kg^-1^)				Polluted soil treatment (mg kg^-1^)			
	Root	Stem	Leaf	Shoot	Root	Stem	Leaf	Shoot
*S. nigrum*	0.29 ± 0.04a	0.32 ± 0.04a	0.30 ± 0.02a	0.30 ± 0.02a	15.48 ± 0.39ab	19.45 ± 0.98a	20.34 ± 0.65a	19.81 ± 0.48a
L-404	0.20 ± 0.02cd	0.25 ± 0.01bc	0.23 ± 0.01c	0.20 ± 0.01cd	18.80 ± 0.49a	17.31 ± 0.28b	19.24 ± 1.46ab	18.16 ± 0.64b
Baisite	0.22 ± 0.02bc	0.25 ± 0.01e	0.26 ± 0.02bc	0.22 ± 0.01c	12.74 ± 0.69b	19.95 ± 0.81a	20.69 ± 0.46a	20.27 ± 0.33a
Zeibutou	0.14 ± 0.02e	0.14 ± 0.02d	0.31 ± 0.06a	0.18 ± 0.02d	18.80 ± 0.66a	16.11 ± 0.26b	18.33 ± 1.39bc	17.06 ± 0.41c
Ziyu	0.16 ± 0.04de	0.29 ± 0.02ab	0.28 ± 0.02ab	0.25 ± 0.01b	18.87 ± 0.90a	15.93 ± 0.59b	17.26 ± 0.26c	16.32 ± 0.35c
Liaoyuanduoli	0.25 ± 0.01ab	0.32 ± 0.02a	0.27 ± 0.01abc	0.26 ± 0.01b	18.54 ± 4.98a	16.21 ± 1.42b	18.29 ± 1.24bc	17.15 ± 1.30bc
L-402	0.12 ± 0.01e	0.30 ± 0.01a	0.28 ± 0.01ab	0.26 ± 0.01b	19.44 ± 1.89a	13.90 ± 0.84c	15.14 ± 0.26d	14.36 ± 0.47d
Meihao	0.25 ± 0.03ab	0.13 ± 0.03d	0.24 ± 0.01bc	0.14 ± 0.01e	19.37 ± 4.96a	16.60 ± 0.81b	18.56 ± 0.79bc	17.25 ± 0.30bc

Data in each column of root, stem, leaf, and shoot of plant species marked by the same letters are not significantly different at *p* < 0.05.

The Cd concentration (mg kg^-1^) differences in various organs of different plants in PS treatment were greater. The Cd concentrations in the stem of *S. nigrum* and Baisite were the highest (*p* < 0.05); the Cd concentrations in the leaves of *S. nigrum*, L-404, and Baisite were the highest (*p* < 0.05). The Cd concentration in the shoots of Baishite and *S. nigrum* were the highest.

### EF and TF of all test plants

**Figure 1:**
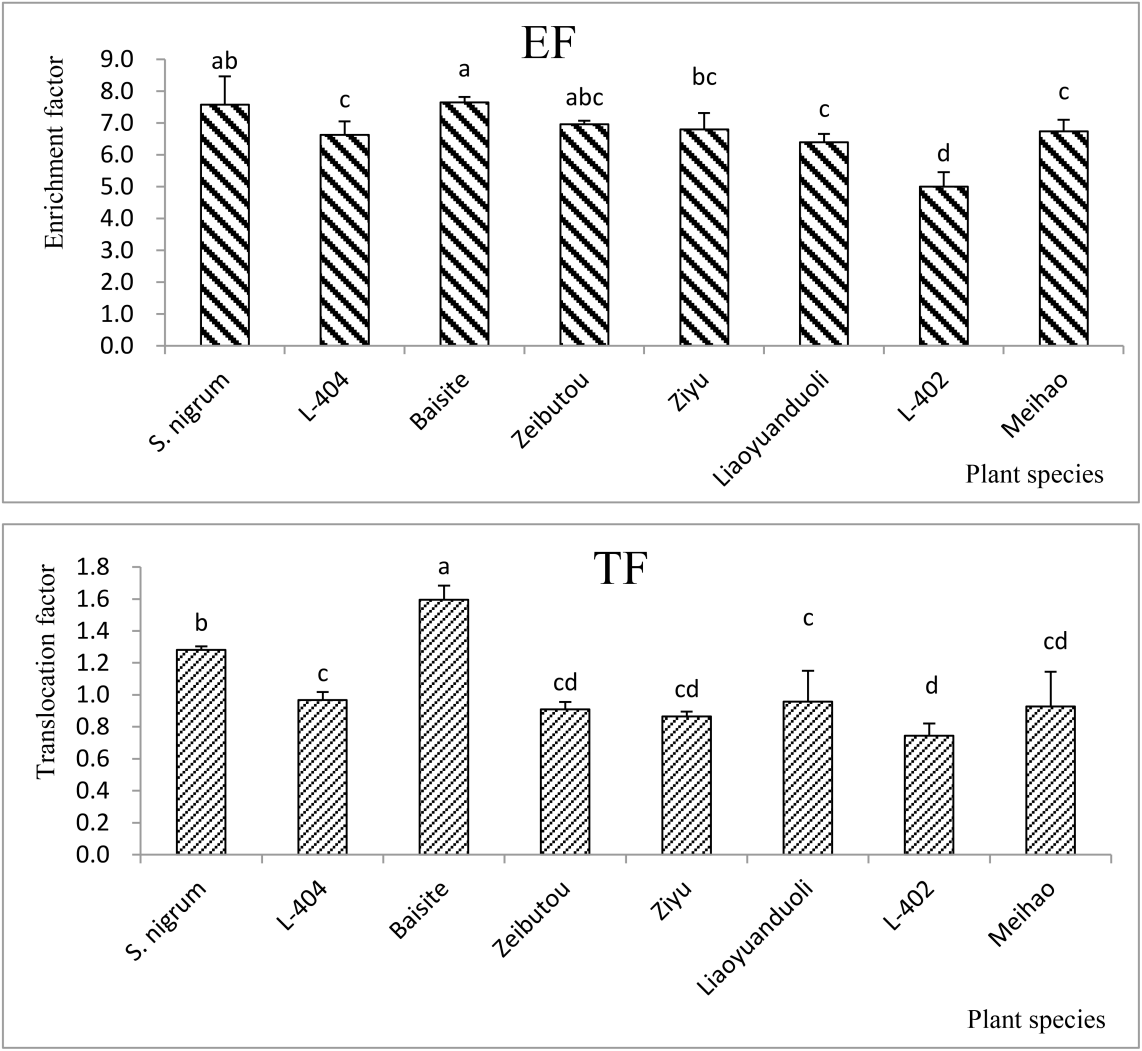
EFs and TFs of all tested *Solanum* plants. Data in each column of plant species marked by the same letters are not significantly different at *p* < 0.05.

Under the PS treatment, the EFs (the Cd concentration ratio of shoots to soils) of all plant species were higher than 1, and the EFs of *S. nigrum* and Baisite were the highest (7.49–7.57), followed by the values obtained in Ziyu (7.37) (**Figure 1**). Other EFs of *S. lycopersicum* cultivars ranged from 5.04 to 6.99.

There were only two TFs (the Cd concentration ratio of shoots to roots) higher than 1, i.e., *S. nigrum* (1.28) and Baisite (1.56). The values of other TFs of *S. lycopersicum* cultivars were from 0.75 to 0.98 (**Figure 1**).

### Biomass differences among different plants

**Figure 2:**
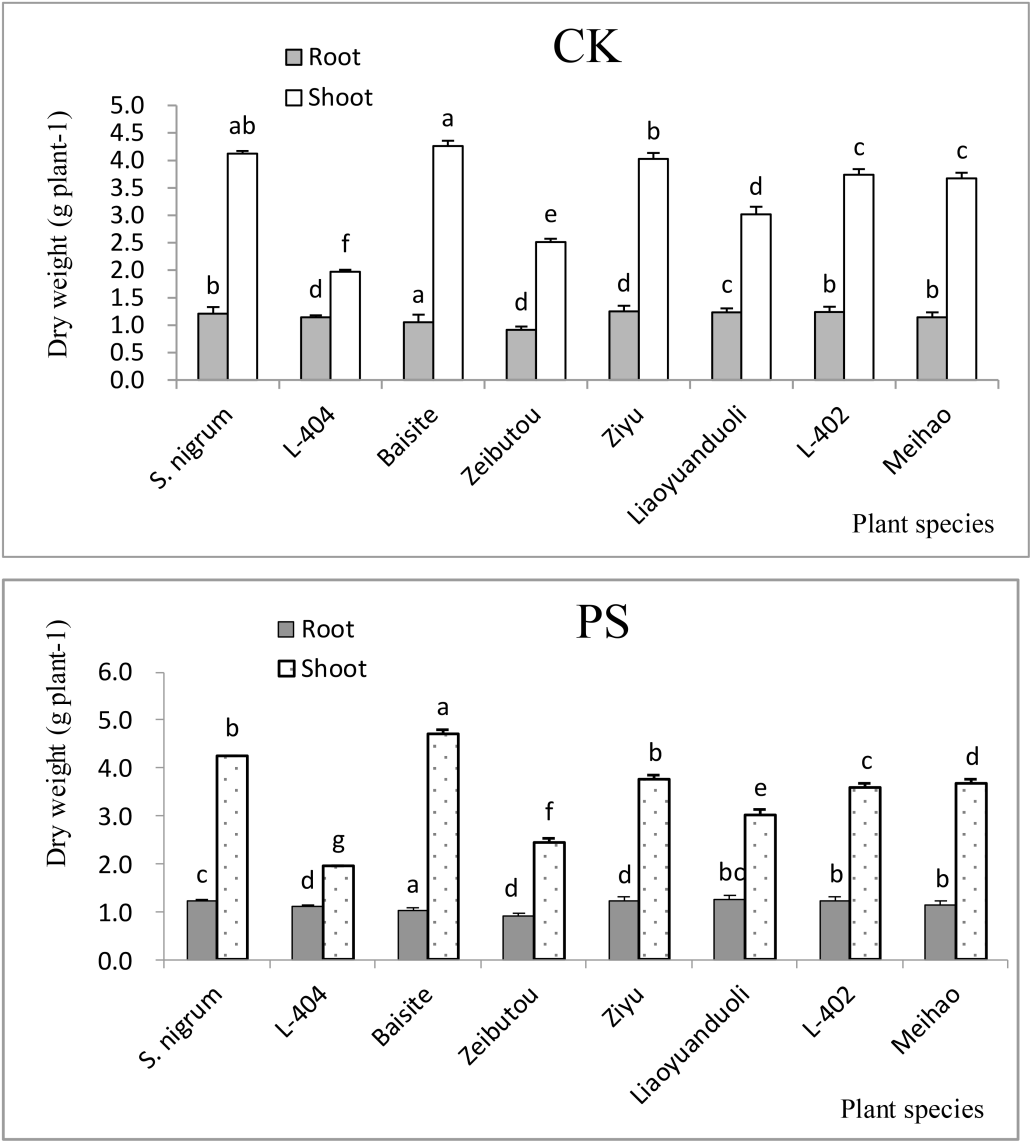
Biomasses of plants. Data in each column of plant species marked by the same letters are not significantly different at *p* < 0.05.

The root and shoot biomasses differences in the CK treatments of different plants were not big (*p* < 0.05) compared to PS treatments (**Figure 2**). The change trend of root biomass was the same as that aboveground (shoots) (**Figure 2**). However, there are significant differences between different plant species treated with CK and PS. The aboveground biomass of Baisite was the highest. *S. nigrum* was the second and L-404 was the lowest (*p* < 0.05). The aboveground biomass of Baisite was 10.4% higher than that of *S. nigrum* and 54.3% higher than L-404. Among them, there were other biomass data of *S. lycopersicum* cultivars (**Figure 2**).

### Cd accumulation capacity in different plants

**Figure 3:**
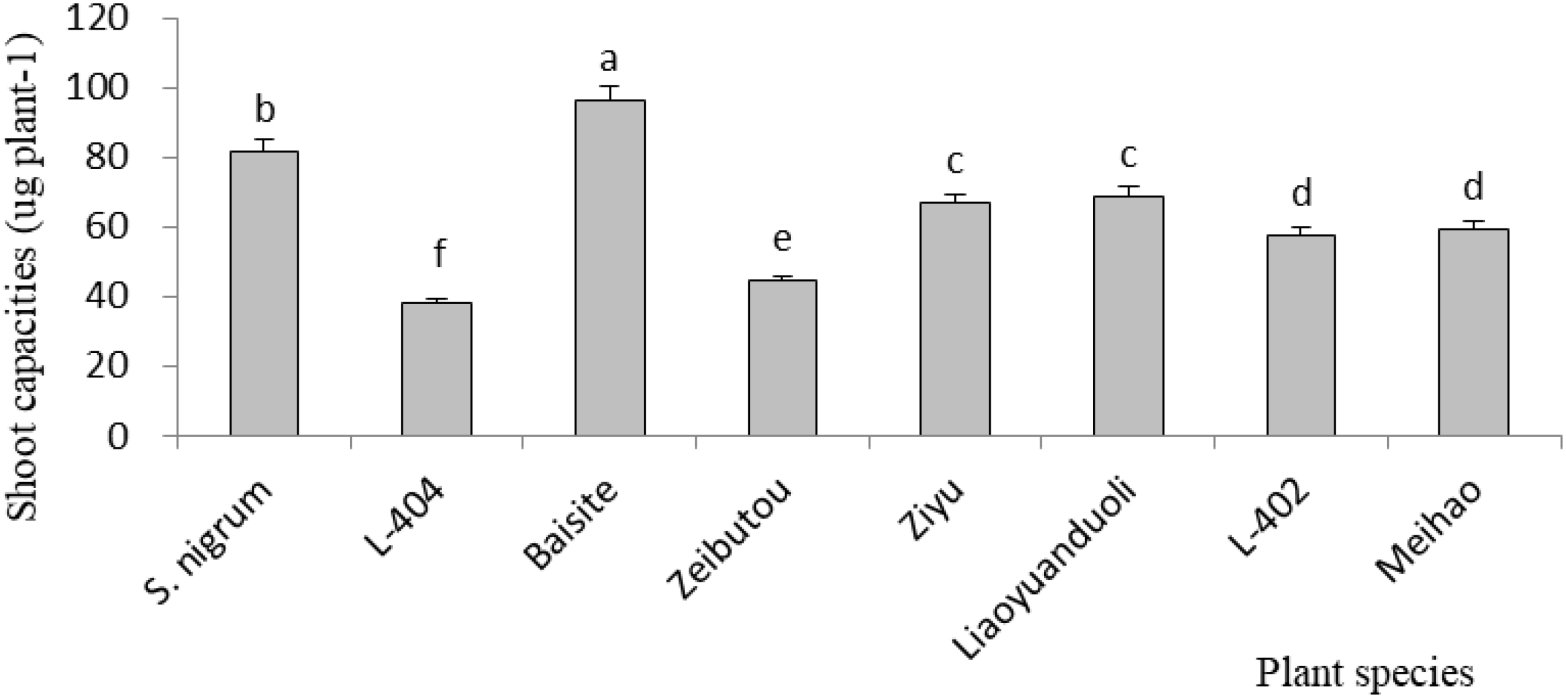
Cd accumulation capacities of aboveground parts. Data in each column of plant species marked by the same letters are not significantly different at *p* < 0.05.

As shown in **Figure 3**, the highest aboveground Cd accumulation was that of Baisite (96.1 µg plant^-1^), and it was 17.6% higher compared to the second Cd capacity of *S. nigrum* shoots (81.7 µg plant^-1^) (*p* < 0.05). The others were all smaller than that of *S. nigrum.*

### Changes in extractable Cd concentrations and physiological parameters

**Figure 4:**
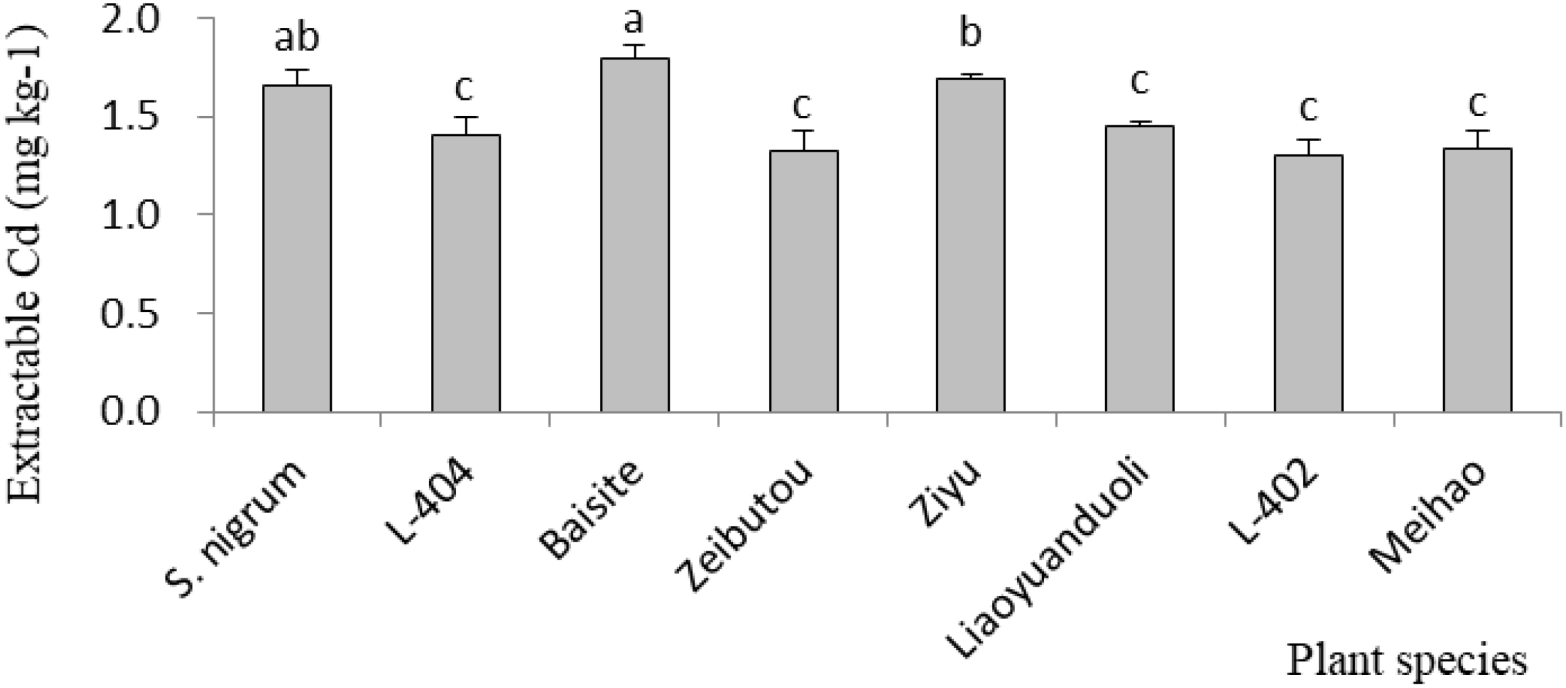
Cd content of extractable species in soil. Data in each column of plant species marked by the same letters are not significantly different at *p* < 0.05.

The ECCs in soils in PS pots are demonstrated in **Figure 4**. The greatest ECCs were those of *S. nigrum*, Baisite, and Ziyu. The ECCs of other *S. lycopersicum* cultivars were essentially the same.

**Figure 5:**
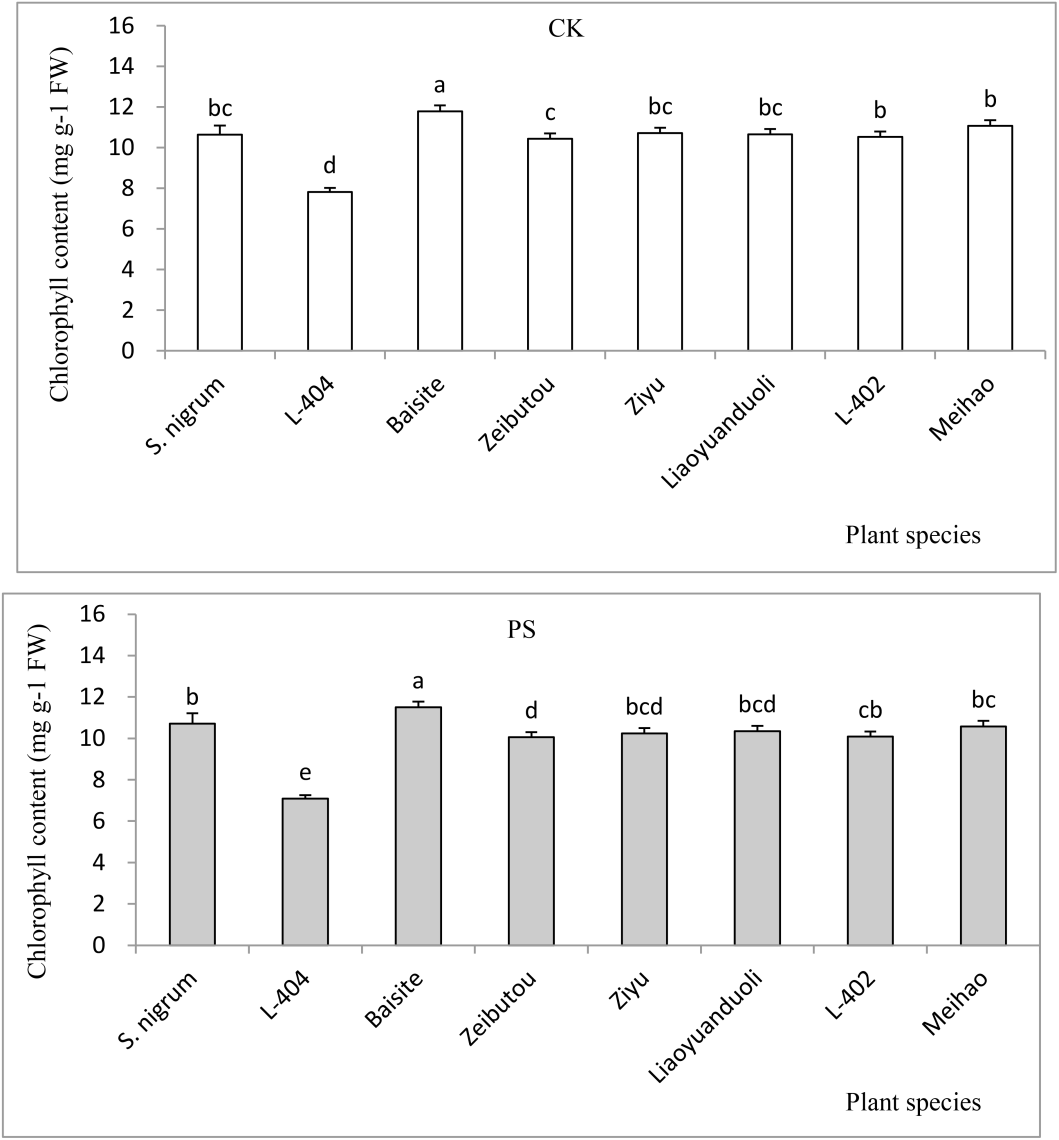
Chlorophyll contents of test plants. Data in each column of plant species marked by the same letters are not significantly different at *p* < 0.05.

The chlorophyll contents in CK and PS treatments are shown in **Figure 5**. The chlorophyll contents of all plants in CKs were basically the same as those in PS treatments (*p* < 0.05). The chlorophyll concentrations of Baisite was the greatest (*p* < 0.05), while the *S. nigrum* chlorophyll contents were the second (*p* < 0.05). The chlorophyll concentrations of other *S. lycopersicum* cultivars were smaller than the previous ones, with Ziyu, Liaoyuanduoli, L-402, and Meihao cultivars being the third and similar to each other (*p* < 0.05); the chlorophyll concentration of L-404 was the lowest (*p* < 0.05) (**Figure 5**).

**Figure 6:**
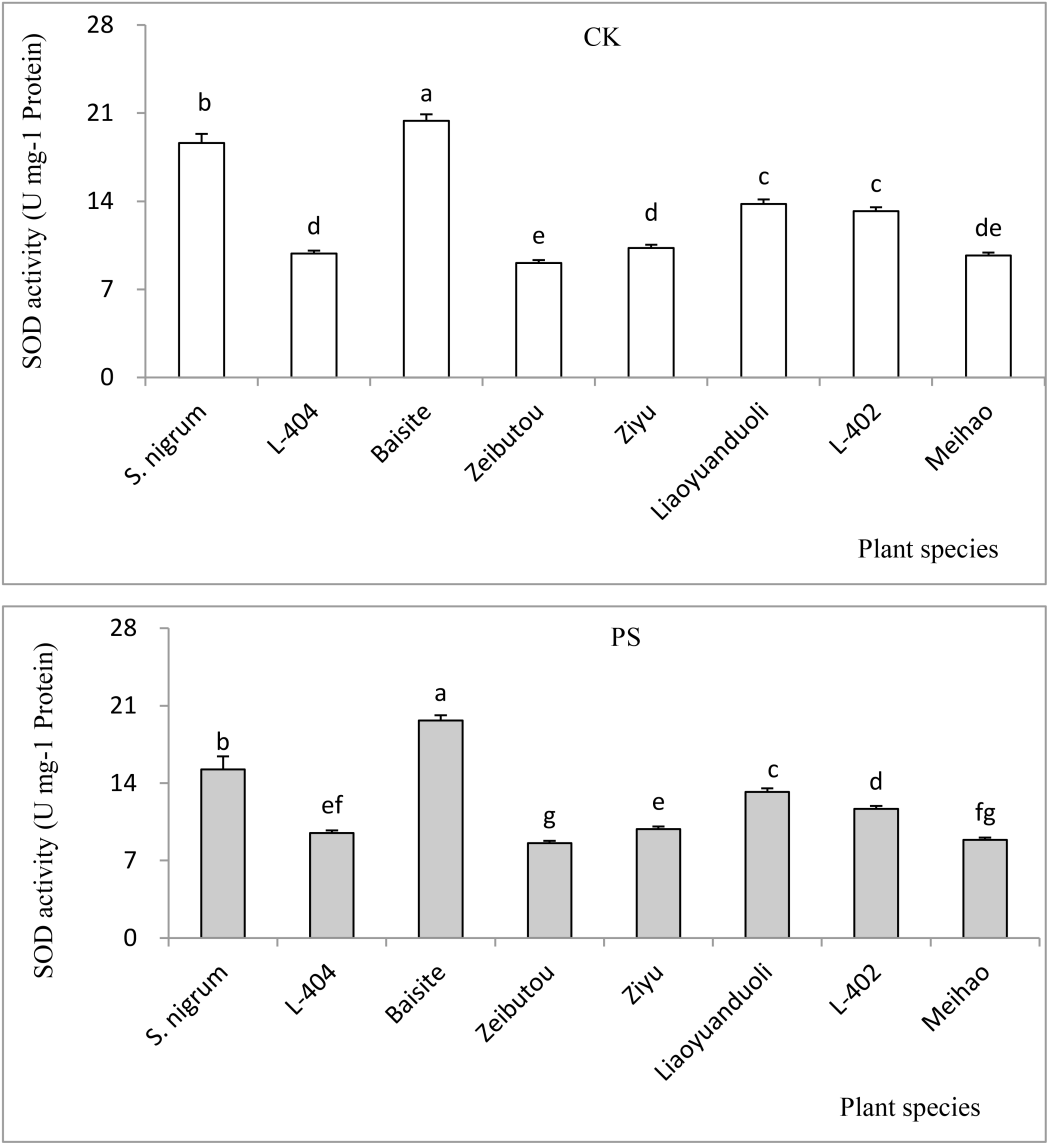
SOD activities of different plants. Data in each column of plant species marked by the same letters are not significantly different at *p* < 0.05.

The SOD values of plants in the CK and PS treatments are presented in **Figure 6**. The SOD data in CK were basically the same as those in the PS treatments (*p* < 0.05). The SOD of Baisite was the highest, with *S. nigrum* being the second. The SOD activities of Zeibutou and Meihao were the lowest (*p* < 0.05) (**Figure 6**).

## Discussion

Heavy metal pollution, especially Cd pollution, has become a major obstacle to the sustainable development of China’s agriculture (Wei et al., 2009). Fortunately, the safe production of agricultural products with moderate and slight soil pollution has attracted more and more attention (Wei and Twardowska, 2013). Another key to screening and identifying Cd-accumulating plants is to develop a practical scientific method system. Environmental factors can change the activity and availability of heavy metal Cd in soil, thereby affecting the accumulation of Cd by plants. It is considered to be another index about the absorption and enrichment of Cd by crops besides genotype (Wei et al., 2009). The EFs of all *S. lycopersicum* cultivars in the PS treatments were all higher than 1 (**Figure 1**). However, the TFs of only *S. nigrum* and the cultivar Baishite were higher than 1 (**Figure 1**). Furthermore, the biomasses of all tested plant cultivars in the PS treatments were not significantly decreased compared to CKs (**Figure 2**). These data indicated that *S. nigrum* as well as the cultivar Baishite showed basic Cd hyperaccumulator characteristics (Wei et al., 2009; Wei and Twardowska, 2013). Obviously, *S. lycopersicum* cultivar Baishite showed the most optimal Cd hyperaccumulation properties (**Figures 1**, **2**). Al-Lahham et al. (2007) reached a similar conclusion as well and demonstrated the suitability of one cultivar of *S. lycopersicum* for the stabilization of sewage sludge.

Usually, heavy metal concentration extracted by plants was increased with their extractable content in rhizospheric soil (Dou et al., 2022). The extractable Cd concentrations in soils of *S. nigrum* and Baishite treatment were the highest (**Figure 4**), but the Cd concentration in Baishite was statistically similar to those in *S. nigrum* (**Table 1**). The chlorophyll contents and biomasses of Baishite were the highest (**Figures 2**, **5**). Their Cd concentration was the highest (**Table 1**). SOD scavenges these ROS in order to protect plant cells and avoid or reduce the damage to plants by stress (Dai et al., 2022).

In general, Cd accumulation capacity (µg plant^-1^) in the shoot of a plant represents its phytoremediation potential due to the main removal mass being equal to the products of its concentration and biomass (Dai et al., 2022). **Figure 3** shows that Cd (µg plant^-1^) was the highest in the cultivar Baishite shoot. This was due to the fact that Baishite had the highest biomass (**Figure 2**). A higher biomass can also cause high Cd accumulation due to the growth force and greater efficiency of antioxidant enzymes (Wei et al., 2018). There were no positive relationships between extractable Cd content in soil, chlorophyll content and SOD activities in leaves, and Cd concentration in shoots and roots (**Table 1**; **Figures 4**-**6**), which indicated that some other mechanisms could play more important roles in Cd accumulation differences between various cultivars. Obviously, some unknown genes concerning Cd accumulation differences among different cultivars may be more important, which might be a future research direction.

## Conclusions

Among the seven tested tomato cultivars, the cultivar Baishite showed basic Cd hyperaccumulation properties. Moreover, cultivar Baishite showed the highest biomass and the highest Cd capacity in shoots. These results indicated that the Cd level did not limit the growth of *S. nigrum* and *S. lycopersicum*. No significant differences between CK and PS were found in the other index. It is necessary to search for more *S. lycopersicum* species with greater Cd accumulation capacity and explanation of the mechanisms of Cd accumulation.

## Data Availability

The original contributions presented in the study are included in the article/supplementary material. Further inquiries can be directed to the corresponding author.
